# Investigation of biocidal efficacy of commercial disinfectants used in public, private and workplaces during the pandemic event of SARS-CoV-2

**DOI:** 10.1038/s41598-022-09575-1

**Published:** 2022-03-31

**Authors:** Annalisa Ambrosino, Concetta Pironti, Federica Dell’Annunziata, Rosa Giugliano, Annalisa Chianese, Giuseppina Moccia, Francesco DeCaro, Massimiliano Galdiero, Gianluigi Franci, Oriana Motta

**Affiliations:** 1 Department of Experimental Medicine, University of Campania “Luigi Vanvitelli”, Via S. Maria di Costantinopoli, 16 80138 Naples, Italy; 2 Department of Medicine Surgery and Dentistry, University of Salerno, via S. Allende, 84081 Baronissi, SA Italy

**Keywords:** Microbiology, Health occupations

## Abstract

This study investigated the performance of 24 commercial disinfectants present on the market during last year according to the manufacturer’s instructions. Recently, national and international organizations of public health performed studies on disinfection products due to the increasing awareness of the potential and growing risks on human health, such as skin damage and reactions in the mucosal lining, especially for the healthcare workers in their frequent daily use. However, there are many limitations in the common cleaning/disinfection products on market as in the selection of effective disinfectants to decontaminate inanimate surfaces. We analyzed the disinfection power of hydrogen peroxide, quaternary ammonium compounds, alcohols, phenols and aldehydes used as active principles according to international guidelines. The antimicrobial properties were assessed by broth microdilution, and antibiofilm properties against *Escherichia coli* (*E. coli*) and *Staphylococcus aureus* (*S. aureus*); their virucidal efficacy was tested against Herpes simplex virus type 1 (HSV-1) and Severe acute respiratory syndrome coronavirus 2 (SARS-CoV-2). The quaternary ammonium compounds demonstrated better efficacy than others and in some cases ready to use products had also virucidal and antimicrobial activities after dilution at 0.125%. The scientific evidence indicates that many commercial products are used at high concentrations and high doses and this could have deleterious effects both on human health and the environment. A lower concentration of active ingredients would avoid the excessive release of chemicals into the environment and improve skin tolerance, ensuring the health and safety protection of workers, including the healthcare operators at their workplace.

## Introduction

In the last year, the Severe acute respiratory syndrome coronavirus 2 (SARS-CoV-2) increased the use of various disinfectants to control the spread of the virus. The World Health Organization (WHO) and Italian Health Institute (Istituto Superiore di Sanità) released many guidelines for disinfectant utilization against the virus, practical tips for cleaning private and public environments (such as schools, offices, healthcare structures, etc.) and protocols to avoid the spread of infection in particular environments. In order to contain the health emergency, guidelines were developed according to hand hygiene, effective disinfection of surfaces and environments for operators, workers and the public. However in many processes additional factors are not taken into accounts such as the characteristics and properties of disinfectants, contact time on the surface, chemical-physical characteristics of surfaces, and possible interactions with disinfectants. One of the most important disadvantages of using a high quantity of disinfectants is the operator sensitisation and related health problems, such as skin irritation and/or dermatitis^1–3^. Irritant contact dermatitis (ICD) is the most common form of occupational skin disease among hospital cleaning workers and it has been reported that mixtures made of detergents/alcohol-based disinfectants cause lower skin irritations than detergent alone^4^; moreover combination of propanols (2-propanol 45% w/w and 1-propanol 30% w/w) showed better skin compatibility compared with the hand rub itself, thanks to the skin hydration function of glycerin^5^ and this, in turn, enhances the compliance of correct hand disinfection in healthcare workers^6^. The lack of necessary training programs for the public, private and workers could raise adverse effects of disinfectants on human health. Homecare people or public cleaning staff, including healthcare workers, very often underestimate their exposure or may lack knowledge regarding the potential harmful disinfectants active compounds, therefore national surveillance of health outcomes related to cleaning and disinfection products should be considered. Moreover, cleaning procedures have been recognized as an occupational risk factor for asthma among healthcare workers, because of their sensitizing or irritant properties, including quaternary ammonium compounds, ethanolamines, chlorhexidine, glutaraldehyde, phenols, peroxy and chloro products^7–10^.

For this reason, it is necessary to provide deep information on the product types available on market, their efficacy on potentially infected surfaces, and information on hazards, legislation and conditions for correct use.

Disinfectants, such as alcohols, aldehydes, hydrogen peroxide, phenols, and quaternary ammonium compounds were recommended by authorities to be used alone or in combinations for disinfection of all surfaces for public health; but it is necessary to evaluate all the negative and positive aspects of specific use, possible production of toxic byproducts, their environmental and human health impacts^11,12^. During last year, detergents, disinfectants and antibiotics were overused for the coronavirus disease control and treatment, and their concentration and dose definitely increase in environments. The ECDC (European Centre of Disease Prevention and Control) suggested the use of sodium hypochlorite solution 0.1% w/w on hard surfaces and always after cleaning with water/detergent solution, while in many cases workers and people used an higher concentration of active substances. The high concentration of sodium hypochlorite could increase the concentration of halogenated byproducts in the environment with toxic effects. Disinfectants and their by-products were frequently detected in surface waters, groundwaters, soils, sediments and wetlands therefore the environmental impacts become a worldwide concern^13,14^.

In a study on two coronaviruses, a comparison of different disinfectant agents showed that ethanol solution at 70% was more effective on the two coronaviruses (mouse hepatitis virus and transmissible gastroenteritis virus) after one minute of contact on hard surfaces than sodium hypochlorite solution at 0.06% v/v^13^.

Human viruses such as SARS coronavirus were destroyed by disinfectants such as sodium hypochlorite solution at 0.1% or ethanol solutions at 71% or hydrogen peroxide after one minute of exposure^14^. However, the logarithmic reduction obtained with ethanol under experimental conditions is not always in line with European standards (EN standards), which require a logarithmic reduction > 4. Other biocides solutions, such as benzalkonium chloride solution between 0.05 and 0.2% or chlorhexidine digluconate solution at 0.02%, were less effective. In practice, label instructions are not always followed and disinfectant products are overused for Sars-Cov-2 virus control since the effective active substances are not yet specifically tested against this virus^15–17^.

The aim of this study is a brief analysis of commercial "disinfection" products, starting with the kinds of products available on the market, on their efficacy according to the conditions for correct use reported under label described and off-label condition to estimate the minimal albeit sufficient concentration to use in decontamination process and avoid overuse of active substances. Disinfectants that are inappropriately used result in both minor and dangerous life-threatening side effects such as skin irritation and damage; they will eventually be emitted in environments, either directly or indirectly, and could be considered important pollutants in the next future.

## Methods

### Materials

All formulations of disinfectants were purchased from commercial suppliers. For each disinfectant, the product composition and the authorised use concentration expressed as product dilution (%) and equivalent active ingredients (mg/L), are shown in Table 1.

**Table 1:** Concentrations of active ingredients in the 24 disinfectants tested at the official use concentration.

Disinfectant	Class of disinfectant	Product composition for 100 g	Official use concentration (%)	Active ingredients concentrations at use concentration (g L^−1^)
1	Alcohol	Ethanol 70 g	Ready to use	700
2	Quaternary ammonium	Limonene 0,002 g benzalkonium chloride 0,5 g	Ready to use	Limonene 0.02 g benzalkonium chloride 5 g
3	Quaternary ammonium	Benzalkonium chloride 1,0 g	Ready to use	10,0
4	Alcohol	Propan-2-olo 6 g	1%	0,6
5	Alcohol	Ethanol 20 g Propan2-olo 8 g	Ready to use	200 80
6	Quaternary ammonium	Didecyl-dimethyl ammonium chloride 0,125 g	Ready to use	1,25
7	Phenols	Ethanol 60 g Propan-2-olo 3 g Biphenyl-2-olo 1 g	Ready to use	600 3 1
8	Quaternary ammonium	Didecyl-dimethyl ammonium chloride 6 g, 2-aminoethanol2 g	1%	0,6 0,2
9	Quaternary ammonium	Didecyl-dimethyl ammonium chloride 0,07 g	Ready to use	Didecyl-dimethyl ammonium chloride 0,7
10	Quaternary ammonium Phenols	Benzalkonium chloride 0,08 g 2-biphenylol 0,03 g	Ready to use	Benzalkonium chloride 0,8 2-biphenylol 0,3
11	Quaternary ammonium	Didecyl-dimethyl ammonium chloride 0,05 g	Ready to use	Didecyl-dimethyl ammonium chloride 0,5
12	Quaternary ammonium	Didecyl-dimethyl ammonium chloride 0,27 g	Ready to use	2,7
13	Quaternary ammonium	Didecyl-dimethyl ammonium chloride 7 g	1%	0,7
14	Alcohol Aldehyde	2-propanol 45 g Limonene 2 g cinnamaldehyde 10 g	Ready to use	2-propanol 450 Limonene 20 cinnamaldehyde 100
15	Quaternary ammonium	Benzalkonium chloride 1 g Didecyl-dimethyl ammonium chloride 8 g	3%	Benzalkonium chloride 0,3 Didecyl-dimethyl ammonium chloride 2,4
16	Quaternary ammonium	Benzalkonium chloride 10 g	2%	2
17	Quaternary ammonium	Benzalkonium chloride 0,05 g Didecyl-dimethyl ammonium chloride 0,08 g	Ready to use	Benzalkonium chloride 0,5 Didecyl-dimethyl ammonium chloride 0,8
18	Quaternary ammonium	Benzalkonium chloride 5 g	10%	5
19	Alcohol Quaternary ammonium	Ethanol 9 g Didecyl-dimethyl ammonium chloride 0,15 g	Ready to use	Ethanol 90 Didecyl-dimethyl ammonium chloride 1,5
20	Oxidizing agents	Hydrogen peroxide 8,2 g	20%	1,64
21	Quaternary ammonium Alcohol	Benzalkonium chloride 3 g Propan2-olo 1 g	25%	Benzalkonium chloride 7,5 Propan2-olo 2,5
22	Quaternary ammonium Alcohol	Benzalkonium chloride 0,75 g Propan2-olo 0,25 g	Ready to use	Benzalkonium chloride 7,5 Propan2-olo 2,5
23	Alcohol	Ethanol 84 g	Ready to use	840
24	Quaternary ammonium	Benzalkonium chloride 5 g Didecyl-dimethyl ammonium chloride 1 g	Ready to use	Benzalkonium chloride 50 Didecyl-dimethyl ammonium chloride 10

### Bacteria/viral strains and cell culture conditions

The bacterial strains used to assess the antibacterial activity and the biofilm degradation were *E. coli* and *S. aureus*, as representative of Gram-negative and Gram-positive, respectively. All strains were purchased from the American Type Culture Collection (ATCC) (Manassas, USA).In detail, for the antibacterial assays *E. coli* ATCC 11229 and S. aureus ATCC 6538 were used; for the biofilm degradation assays *E. coli* ATCC 25992 and *S. aureus* 25923 were chosen as biofilm producers, while *S. aureus* ATCC 6538 and E. coli ATCC 11229 as non-biofilm producers. HSV-1 (strain SC16) and SARS-CoV-2 (strain VR PV10734, kindly donated by the Lazzaro Spallanzani Hospital of Rome, Italy) were propagated on Vero cell line (ATCC CCL81). Eagle’s Minimum Essential Medium (EMEM) supplemented with 2 mM L-Glutamine, 100 IU/mL penicillin-streptomycin solution, and 10% Fetal Bovine Serum (FBS) were used for the cell growth.

### Determination of minimum inhibitory concentration (MIC)

The antimicrobial activity was conducted following the broth microdilution method, following the guidelines of the National Committee on Clinical Laboratory Standards (NCCLS). In detail, *E. coli* and *S. aureus* were plated on Brain Heart Infusion (BHI) agar plates (Sigma-Aldrich, Missouri, USA) and incubated at 37 °C overnight (O. N.). A fresh colony of both bacteria was inoculated in BHI-broth (Sigma-Aldrich, Missouri, USA) and incubated at 37 °C under vigorous orbital shaking (180 rpm) for 20 h. The following day, 300 µL of bacterial suspension was inoculated in fresh BHI-broth and incubated at 37 °C at 180 rpm until mid log-phase growth (6 × 10^8^ colony-forming units (CFU/mL). Three serial dilutions were performed to obtain a final bacterial concentration of 1 × 10^6^ CFU/mL. Fifty microliters of bacterial inoculum were added to each well of a sterile 96-well plate. Meanwhile, the 24 compounds were subjected to serial dilutions in phosphate buffered saline 1X (PBS 1X Sigma-Aldrich, Missouri, USA) and 50 µL of each dilution was added to the wells. Ampicillin (10 µg/mL) and vancomycin (5 µg/mL) were used as positive controls for *E. coli* and *S. aureus,* respectively. The plates were incubated at 37 °C at 180 rpm for 20 h. Bacterial growth was monitored using a TECAN microplate reader (Sunrise^TM^, Männedorf, Switzerland), at an optical density (OD) of 600 nm^18,19^.

### Biofilm degradation assay

*E. coli* ATCC 25,992 and *S. aureus* 25,923 were grown overnight in LB broth supplemented with 1% glucose and incubated at 37 °C. The overnight culture of *S. aureus* and *E. coli* was diluted at 0,2 OD 600 nm LB supplemented with 1% of glucose. An aliquot of 100 µl of bacterial suspension was seeded in a 96-well plate and incubated under static conditions at 37° O/N to form the mature biofilm. To determine the effect of 24 compounds on mature biofilm, the non-adherent cells were removed with 2 washes of PBS 1x, and 100 µl of each substance at different concentrations were added. The 96-well plates were incubated at 37 °C O/N to allow substances to act on biofilm degradation. Ampicillin and vancomycin were used as positive controls for *E. coli* and *S. aureus*, respectively. Biofilm degradation was evaluated by 0.1% of crystal violet staining. The supernatant was removed to eliminate the cells in suspension, and the wells were gently washed twice with PBS 1x. The biomass biofilm was stained with 100 µl of crystal violet and the plate was incubated at room temperature (RT) for 40 min^16^. After incubation, the crystal violet was solubilized with 100 µl of ethanol. The plate was read at a wavelength of 570 nm and the percentage of biofilm degradation was calculated with the following formula.

$$\% {\text{Biofilm degradation}} = 1-({\text{OD570 of the test sample}}/{\text{OD570 of negative control}}) \times 100.$$

### Plaque assay

The potential antiviral effect of 24 commercial disinfectants was assessed with the plaque assay method against HSV-1 and SARS-CoV-2. A drop (5 µL) of different concentrations of each disinfectant (as shown in the graph) was allowed to evaporate together with 5 µL virus (1 × 107 PFU) on a sterile plastic surface. After 1 hour, the evaporated mixture was collected, diluted to infect a Vero cells monolayer at 0.01 MOI/cell and incubated for 1 h to provide the viral entry. After the adsorption time, the monolayer was washed with PBS 1X, overlaid with EMEM supplemented with 3% carboxymethylcellulose and incubated for 48 h at 37 °C. At the end, the cells were fixed with 4% formaldehyde and stained with 0.5% crystal-violet. The experiments were performed in triplicate. Cells infected with the untreated virus were used as a negative control. The percentage of viral inhibition was estimated by counting the number of plaques obtained compared to the negative control. The concentrations of disinfectants that inhibited the plaque formation by 90% (IC90) were determined.

### Data analysis

All the experiments were performed in independent triplicate and results were expressed as mean ± standard deviation. Statistical analysis was performed by using ANOVA: P-value, R-square and F value were calculated to determine the significance of the results. Statistical analyses were performed with GraphPad Prism 7.0 (GraphPad Software, Inc., San Diego, CA). The results were considered significant at P-value < 0.05 and R-square > 0.8. The datasets used and/or analysed during the current study are available from the corresponding author on reasonable request.

## Results

### Disinfectant antibacterial activity

The antibacterial activity of 24 currently commercially available compounds was tested against *E. coli* and *S. aureus* (Table 2). A similar trend was detected for D 3–5-6–7-9–15-16–17 with a MIC-value of 0.02, 1.25, 0.002, 20, 0.001, 0.01, 0.004, 0.002, g/L, respectively, against both bacterial species. For D 2–19–20 the inhibition growth up to 0.005 and 0.01 g/L was recorded, against *S. aureus* and *E. coli,* respectively. Versus *E. coli*, D 18–21–22 showed activity up to 0.03 g/L, while against *S. aureus*, D18 and 21 recorded a MIC-value of 0.06 g/L and D22 inhibited up to 0.01 g/L. Greater effectiveness was verified in D 8 and 13 towards *E. coli* compared to *S. aureus*, inhibiting the bacterial growth up to 0.01 and 0.02 g/L, respectively. Otherwise, D 4–10–12–14 were more effective against Gram-positive bacteria, recording a MIC-value of 0.05, 0.002, 1 and 0.39 g/L against *S. aureus* in contrast to MIC-value of 0.1, 0.005, 1.25 and 0.78 g/L against *E. coli*. A similar Gram-negative inhibition was shown for D 11 and 24, with inhibition up to 0.2 g/L against *E. coli,* while 0.05 and 0.1 g/L for *S. aureus*. Finally, D1 and D23 represented compounds with lower antibacterial power, recording inhibition efficacy up to 75 and 12.5 g/L against E. coli and up to 10 and 3.12 g/L versus *S. aureus.* The dose–response curve associated with D1–D24 activity against *E. coli* and *S. aureus* were shown in Supplementary Figs. 1 and 2, respectively.

**Table 2:** Antibacterial activity of 24 disinfectants against E. coli ATCC 11,229 and S. aureus ATCC 6538.

Disinfectant	MIC value (g/L)	
	*E. coli*	*S. aureus*
1	75	10
2	0.01	0.005
3	0.02	0.02
4	0.1	0.05
5	1.25	1.25
6	0.002	0.002
7	20	20
8	0.01	0.02
9	0.001	0.001
10	0.005	0.002
11	0.2	0.05
12	1.25	1
13	0.01	0.02
14	0.78	0.39
15	0.01	0.01
16	0.004	0.004
17	0.002	0.002
18	0.03	0.06
19	0.01	0.005
20	0.01	0.005
21	0.03	0.06
22	0.03	0.01
23	12.5	3.12
24	0.2	0.1

### Disinfectant biofilm degradation

The 24 disinfectants were evaluated for the ability to degrade the preformed biofilm of *S. aureus* and *E. coli* through the crystal violet staining. 40% of biofilm degradation was set as cut off, below this value the action was considered irrelevant (not detected). Particularly, for biofilm of *E. coli* only compounds D 1,2,4,11,13,14,16,18,20,21,23,24—at the minimum concentrations (g/L) reported in Table 3, showed 40% biofilm degradation. On the other hand, for the biofilm produced by *S. aureus*, disinfectants had a different action. In detail, only compounds D 6,8,9,12,13,14,17,18,19,20,21,23 have recorded a 40% degradation of the biofilm (Supplementary Materials, Fig. 3–4).

**Table 3:** Biofilm degradation activity of 24 disinfectants against E. coli and S. aureus.

Disinfectant	Threshold line (40% of biofilm degradation) g/L	
	*E. coli*	*S. aureus*
1	350	ND
2	5	ND
3	ND	ND
4	0.15	ND
5	ND	ND
6	ND	0.62
7	ND	ND
8	ND	0.15
9	ND	0.7
10	ND	ND
11	0.01	ND
12	ND	0.1
13	0.7	0.006
14	28.1	28.1
15	ND	ND
16	0.12	ND
17	ND	0.2
18	1.2	2.5
19	ND	22.5
20	0.4	0.2
21	0.9	7.5
22	ND	ND
23	840	105
24	12.5	ND

### Disinfectant antiviral activity

The antiviral activity of 24 disinfectants was explored against DNA and RNA enveloped viruses (HSV-1 and SARS CoV-2, respectively). We tried to mimic a real condition in which a surface disinfectant could be used. In detail, we tested the virucidal activity of the disinfectants on a contaminated surface. Then, to quantify the antiviral power of each compound avoiding its normal cytotoxicity, we infected a cell monolayer, as described in “Biofilm degradation assay”, as soon as the mixture of virus and compounds evaporated.. Basically, the virus was left to interact on a plastic surface with a single drop of each compound at the concentrations reported in Table 4 Then, the mixture was left to evaporate for 1 h. Subsequently, each mixture was diluted to infect the cell monolayer at 0.01 MOI, which was incubated for the time of virus adsorption (1 h for HSV-1 and 2 h for SARS-CoV-2). As shown in Supplementary Materials Fig. 5, the compounds, D 1–2–8–12–13–14–15–16–18–20–21–22–24 were not active against both viruses, while the other disinfectants showed remarkable dose-dependent inhibition of the viral replication. Data showed that most compounds exhibited similar inhibitory activity against HSV-1 and SARS-CoV-2 at the same concentrations. In detail, setting IC90 as the treshold line, D3–5–6–9–10–11–17–19–23 exhibited inhibitory activity with IC90 at 0.025, 12.5, 0.078, 0.043, 0.008, 0.037, 0.008, 0.093, and 210 g/L, respectively. Only two of 24 disinfectants (D4 and D7) showed a different efficacy of inhibition against DNA and RNA viruses. Indeed, D4 interfered with HSV-1 infection with 90% inhibition at 0.075 g/L, and SARS-CoV-2 at 0.037 g/L; while D7 showed better inhibitory activity against SARS-CoV-2 with IC90 at 0.375 g/L compared to HSV-1 with IC90 at 3 g/L.

**Table 4:** Antiviral activity of 24 disinfectants against HSV-1 and SARS-CoV-2.

Disinfectant	IC90 (g/L)	
	HSV-1	SARS-CoV-2
1	/	/
2	/	/
3	0.025	0.025
4	0.075	0.037
5	12.5	12.5
6	0.078	0.078
7	3	0.375
8	/	/
9	0.043	0.043
10	0.008	0.008
11	0.037	0.037
12	/	/
13	/	/
14	/	/
15	/	/
16	/	/
17	0.008	0.008
18	/	/
19	0.093	0.093
20	/	/
21	/	/
22	/	/
23	210	210
24	/	/

## Discussion

Since last year, due to the pandemic of coronavirus disease, the use of disinfectants to prevent microbial infections is rapidly increasing worldwide^20–26^. Numerous studies analyzed the role of safe for humans chemical agents with high bactericidal and virucidal potential^27–31^. However, currently there is still very little knowledge about the specific disinfectant agents for this new virus with a biosafety level-3 (BSL-3). For this reason, international organizations recommended active agents tested against the other non potentially dangerous coronaviruses. However, it’s known that even viruses within the same family could respond differently to a given disinfectant and scientific results disagree about which disinfectants would be more efficaceous against SARS-CoV-2. In our study, we analysed the efficacy of some commercial products available on market to sanitize surfaces and to understand the real efficaceous concentration in use both for antibacterial and virucidal capacity. Quaternary ammonium compounds (QACs) based disinfectants play an important role in veterinary medicine and the control of animal diseases^32–34^. QACs are cationic surface active detergents widely used for the control of microorganisms in clinical and industrial environments. QACs are commonly used as antimicrobial pesticides, even if their biocidal properties were under the spotlight in recent years highlighting also the disadvantages^35,36^. The QACs disadvantages were associated with the presence of long alkyl chains that can induce fatal damage to a wide variety of organisms; they can bind to negatively charged lipid bilayers and lead to dissociation of the cell membrane components and leakage of intracellular components, resulting in cell death^37,38^. In particular, databases for Didecyl dimethyl ammonium chloride (DDAC) and alkyl (C12, C14, C16) dimethyl benzyl ammonium chloride (C12–C16 ADBAC) are complete to support the registered uses of these pesticides. DDAC and C12–C16 ADBAC are permanently charged cationic compounds, and available studies indicate that both DDAC and C12–C16 ADBAC are poorly absorbed via the oral and dermal exposure routes, and are primarily eliminated in feces. ECHA^39^ and EPA^40^ concluded that oral and dermal absorption of DDAC and C12–C16 ADBAC does not exceed 10%. Low dermal and oral absorption of DDAC and C12–C16 ADBAC is consistent with the lack of systemic toxicity observed across available repeated doses from oral and dermal toxicology studies conducted with beagles, mice, and rats. Toxicological findings from acute, subchronic, and chronic oral toxicity studies are consistently characterized by local stomach irritation, reduced food consumption, reduced body weight, and reduced weight gain. Misuse of these preparations may be deleterious to human health and when these chemicals are released through evaporation, they will have toxic and hazardous effects on the environment^41–43^. The most common disinfectant products on the market were formulated with these compounds and the formulations that we have examined showed antibacterial activity against *E. coli* and *S. aureus*. Formulations indicated in Table 1 as D6, D9, D11, D12 contain DDAC and the manufacturer reported on the label a description such as “ready to use product”, without preliminary dilution. However, our data demonstrated the in vitro efficacy of these agents at very low concentrations, against Gram-positive and Gram-negative bacteria. In detail, the efficiency range was 0.001–0.0005 g/L against *S. aureus* and 0.0005 g/L against *E. coli*, respectively for D6 and D9. Whereas similar labeled products D11 and D12 exhibited a lower activity at the same concentration. The different activity was probably attributed to the different formulation compositions and the stability condition of disinfectant products. Therefore, our studies were focused on the comparison between commercial disinfectant formulations in real use. Regarding the results obtained through the broth microdilution method, DDAC showed a similar trend compared to mixtures of benzalkonium chloride and DDAC, albeit with slightly greater efficacy. On the other hand, reported virucidal results showed a similar trend such as all solutions could be considered toxic against viruses to commercial concentration and “ready to use” solution, like D3 and D7, was very toxic against SARS-CoV-2 also at a concentration of 0,125 %. Alcohol based formulations were considered the best solutions for the surface disinfection process, even if in our investigation we obtained different results. In detail, for D1 formulation, ethanol more than 70%, recorded a MIC value lower than QACs compounds and an inhibition growth against *S. aureus* at 5 g/L and 45 g/L against *E. coli*; while isopropyl alcohol (D4) showed a bacterial growth inhibition up to 0.05 and 0.02 mg/L for *S. aureus* and *E. coli*, respectively. For the mixture of both alcohols, a MIC value greater than the individual component was detected, confirming the improved antibacterial efficacy of isopropyl alcohol. In literature the optimum bactericidal concentration of alcohol solution reported is from 60 to 90%, although some studies showed that at a concentration beyond 70% the cell wall is sealed up preventing further entry of alcohol. For this reason, international organizations recommended formulations at 70% alcohol^44,45^. Many studies investigated the best type of alcohol and concentration for the disinfection of stethoscopes^46–49^. Stethoscopes are potential vectors for health care associated infections (HAI) and pose a potential risk in health care settings^50^. The virucidal effects of alcohol were associated with their ability to disintegrate RNA, interfering with membrane integrity, and denaturation of viral proteins. Alcohols are amphoteric compounds and these properties promote the disintegration of the tertiary structure of proteins, causing the breakdown of the intramolecular hydrogen bonds within the structure. Chojnacki et al. 2021 evaluated the performance of 46 commercially hand sanitizers available on market for antibacterial activity toward prototypical Gram-positive (*Staphylococcus aureus*) and Gram-negative (*Escherichia coli*) bacterial pathogens. Phenols and mixtures with alcohol disinfectants played an important role in hospital disinfectants thanks to their antimicrobial and virucidal efficacy^51^. Results confirmed the efficacy of phenol derivatives at a concentration of 0.5 to 5 g/L in a few minutes, as reported for HIV, through denaturing proteins and membrane disruption, which leads to leakage of components.

## Conclusions

The experimental data highlighted the antimicrobial and virucidal activity of all commercial products evaluated on *Escherichia coli* (*E. coli*) and *Staphylococcus aureus* (*S. aureus*) and DNA and RNA viruses, such as HSV-1, and SARS-CoV-2. Additionally, our results demonstrated a better efficiency of QACs than others after dilution at 0,125%. The scientific evidence suggests an overuses of many commercial products inducing possible health effects, such as skin damage to the healthcare workers in their frequent daily use, and growing of environmental contamination. As noted, some disinfectant products appeared effective toward one or both organisms, whereas the antibacterial effects of other sanitizers seemed to wane. Further, there may be minor, yet appreciable, differences in the efficacy of QACs and alcohol-based formulations. Thus, it may be wise to implement formal requirements for efficacy data as a requisite for the continued production of disinfectants that have been introduced to the market under emergency COVID-19 authorization. Results of these studies indicate that antibacterial testing should probably be conducted and performed at lower concentrations to avoid excessive release of chemicals into the environment that can cause serious damage: e.g. modification of the environmental microbiota, potential threat to living beings and ecosystems, pollution of water and groundwater. Moreover, it will improve skin tolerance and consequently reduce occupational skin diseases such as irritant contact dermatitis and skin irritation processes, ensuring the health and safety protection of the operators at their workplace. These outcomes play a significant role in terms of user compliance with disinfection procedures, especially during the current COVID-19 pandemic. Similarly, it may be important to evaluate the effectiveness of sanitizers toward multiple strains of viruses.

## Supplementary Information

Supplementary Information.
