# Supplementary material for: Investigation of biocidal efficacy of commercial disinfectants used in public, private and workplaces during the pandemic event of SARS-CoV-2
Source: Sci Rep. 2022 Mar 31;12:5468. doi: 10.1038/s41598-022-09575-1 (PMC8969816; doi:10.1038/s41598-022-09575-1)
Supplement: Supplementary file 1 — Supplementary Information. [file 41598_2022_9575_MOESM1_ESM.docx]

**Investigation of biocidal efficacy of commercial disinfectants used in public, private and workplaces during the pandemic event of SARS-CoV-2**

# Annalisa Ambrosino^1,+^,Concetta Pironti^2,+^,  Federica Dell’Annunziata^1^, Rosa Giugliano^1^, Annalisa Chianese^1^, Giuseppina Moccia^2^, Francesco DeCaro^2^, Massimiliano Galdiero^1^, Gianluigi Franci^2^, Oriana Motta^2,*^

^1^Department of Experimental Medicine, University of Campania “Luigi Vanvitelli”, Via S. Maria di Costantinopoli 16, 80138 Naples, Italy

^2^Department of Medicine Surgery and Dentistry, University of Salerno, via S. Allende, 84081 Baronissi (SA), Italy

^*^corresponding Author: Oriana Motta [omotta@unisa.it](mailto:omotta@unisa.it)

^+^these authors contributed equally to this work as first authors

**Supplementary materials**

**Figure 1.** Antibacterial activity of 24 disinfectants (A-X) against *E. coli* ATCC 11229 (P-value <0.005). Ampicillin (10ug / mL) was used as a positive control.

**Figure 2.** Antibacterial activity of 24 disinfectants (A-X) against *S. aureus* ATCC 6538 (P-value <0.005). Vancomycin (20ug / mL) was used as a positive control.

**Figure 3.** Antibiofilm activity of 24 disinfectants (A-X) against *S. aureus* ATCC 6538 (P-value <0.005). Vancomycin (20ug / mL) was used as a positive control.

**Figure 4.** Antibiofilm activity of 24 disinfectants (A-X) against *E. coli* ATCC 11229 (P-value <0.005). Ampicillin (10ug / mL) was used as a positive control.

**Figure 5.** Antiviral activity of disinfectants (A-K) against SARS-CoV-2. Rhamnolipids M15RL (50 µg / mL) was used as a positive control.


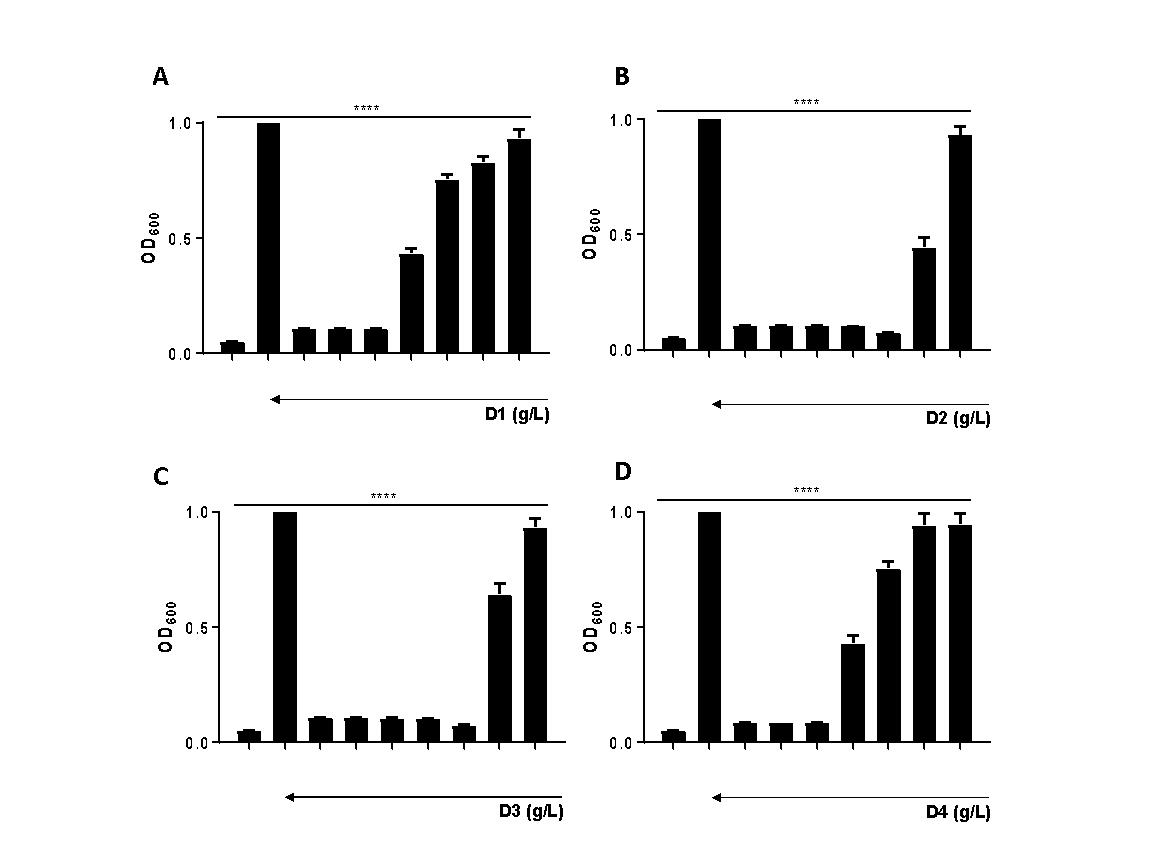

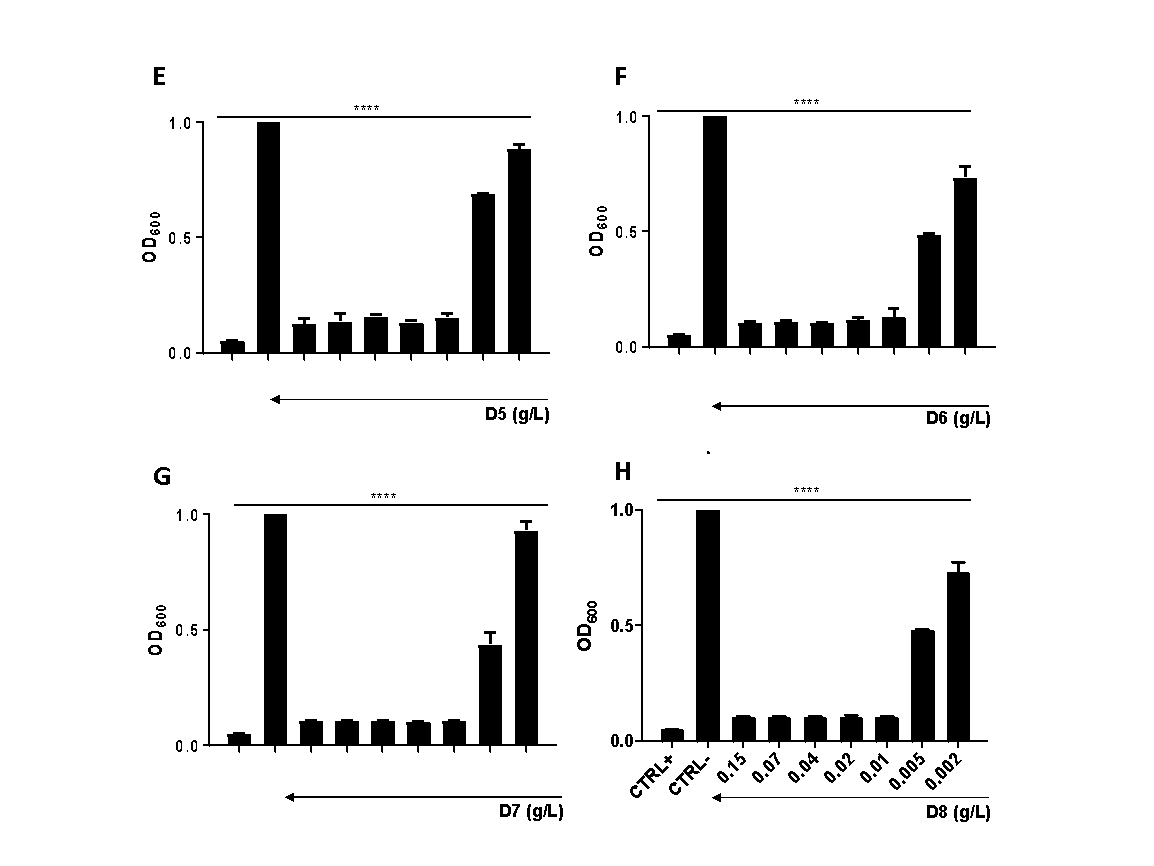

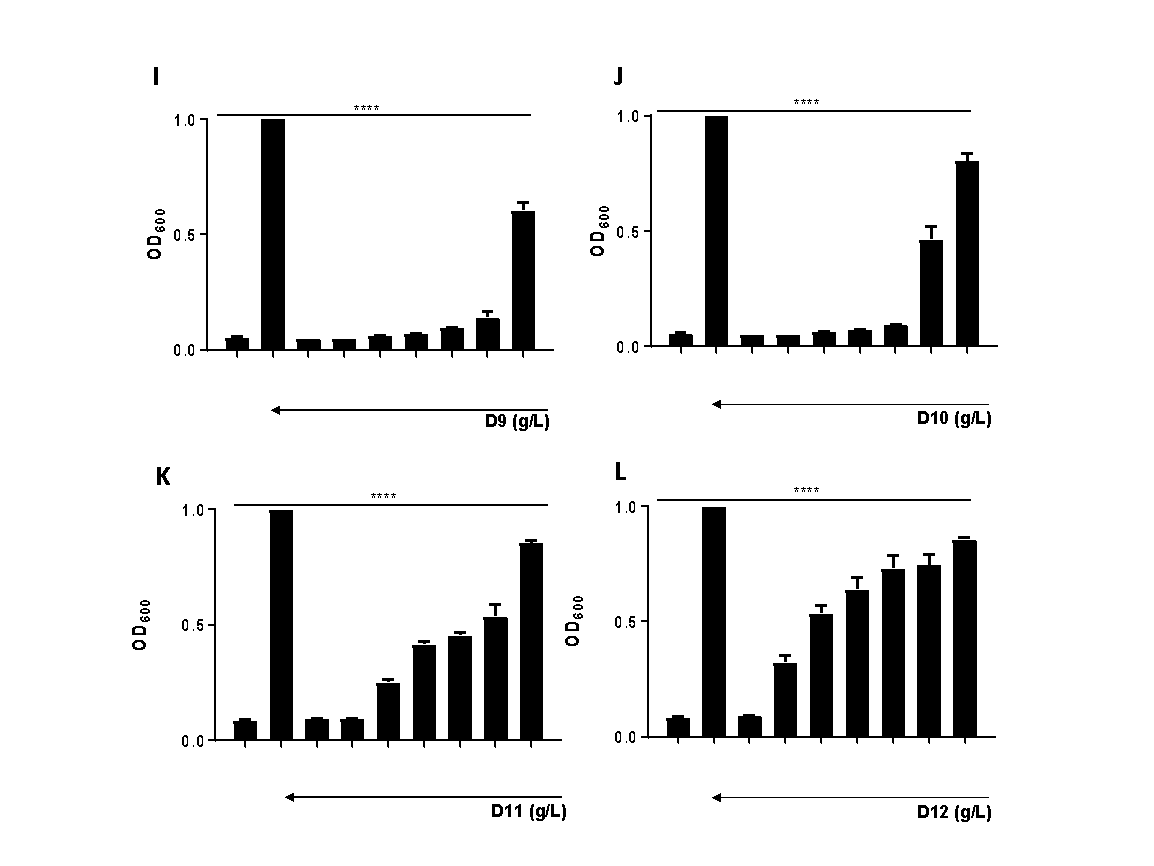

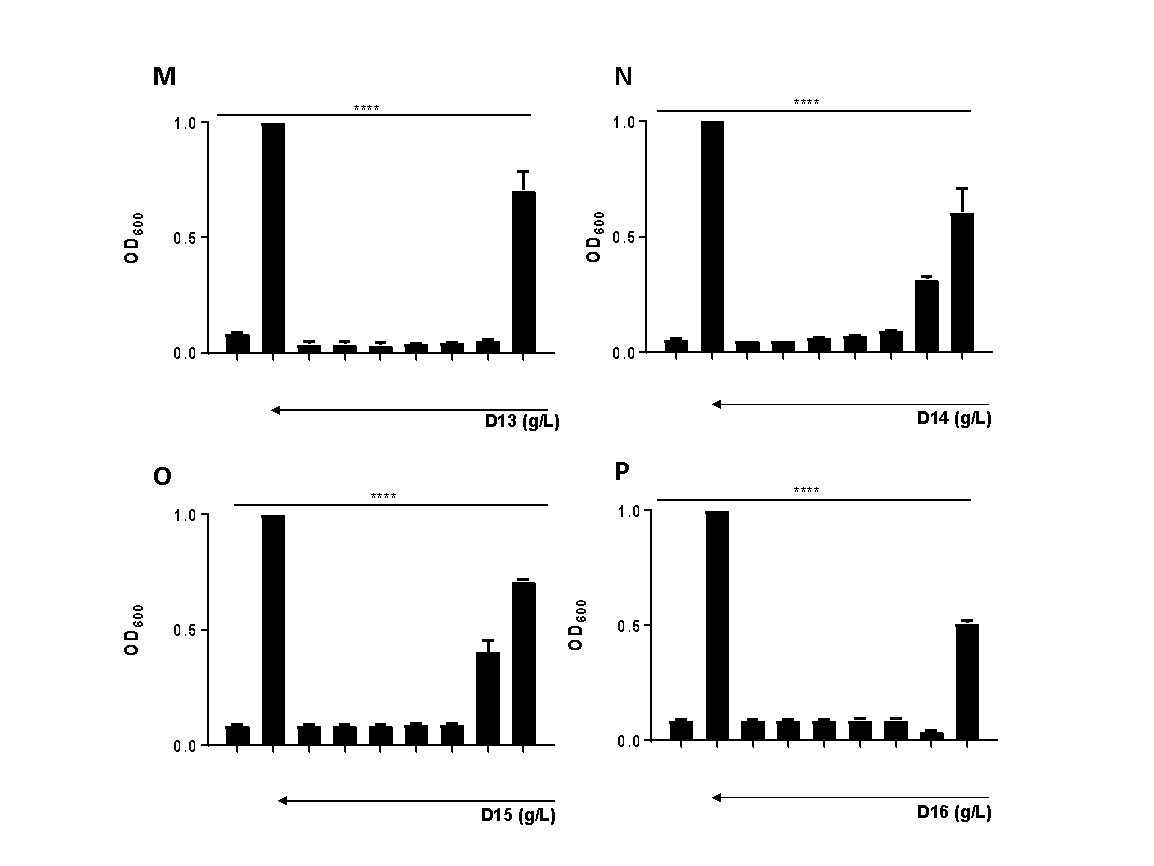

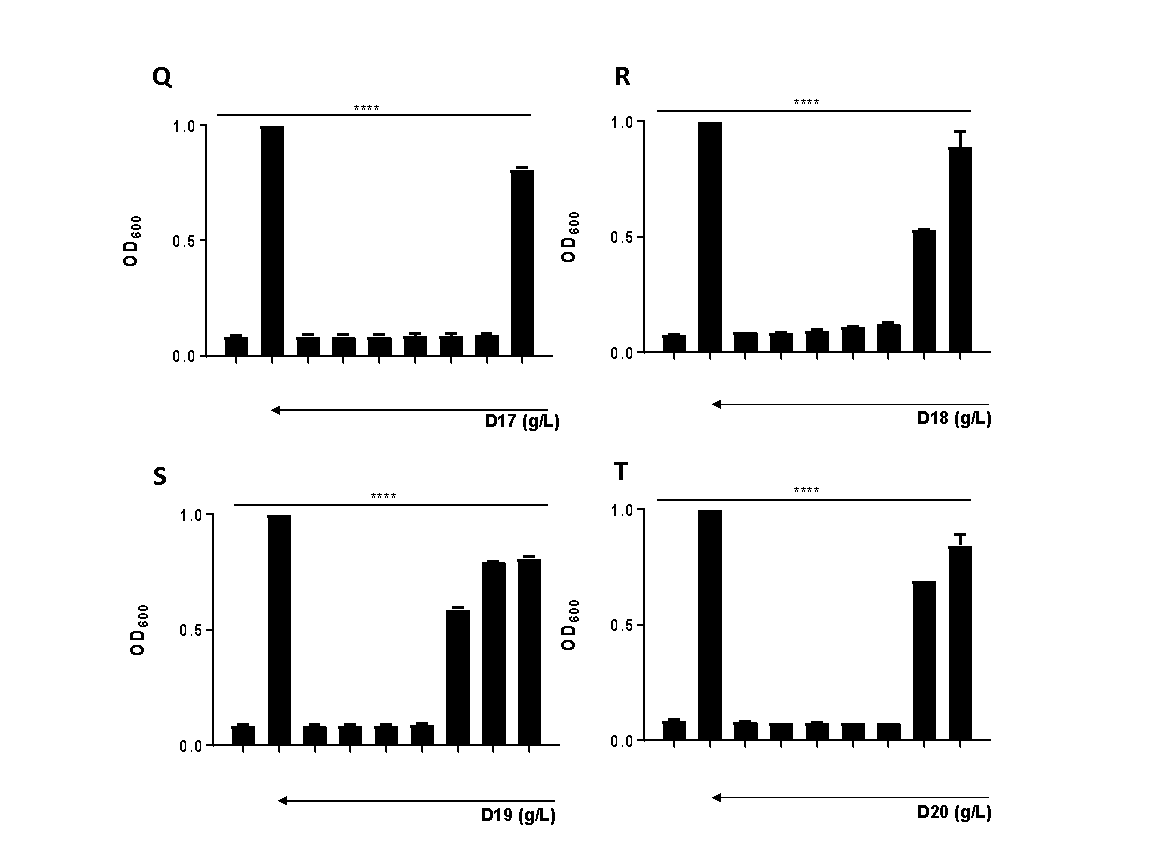

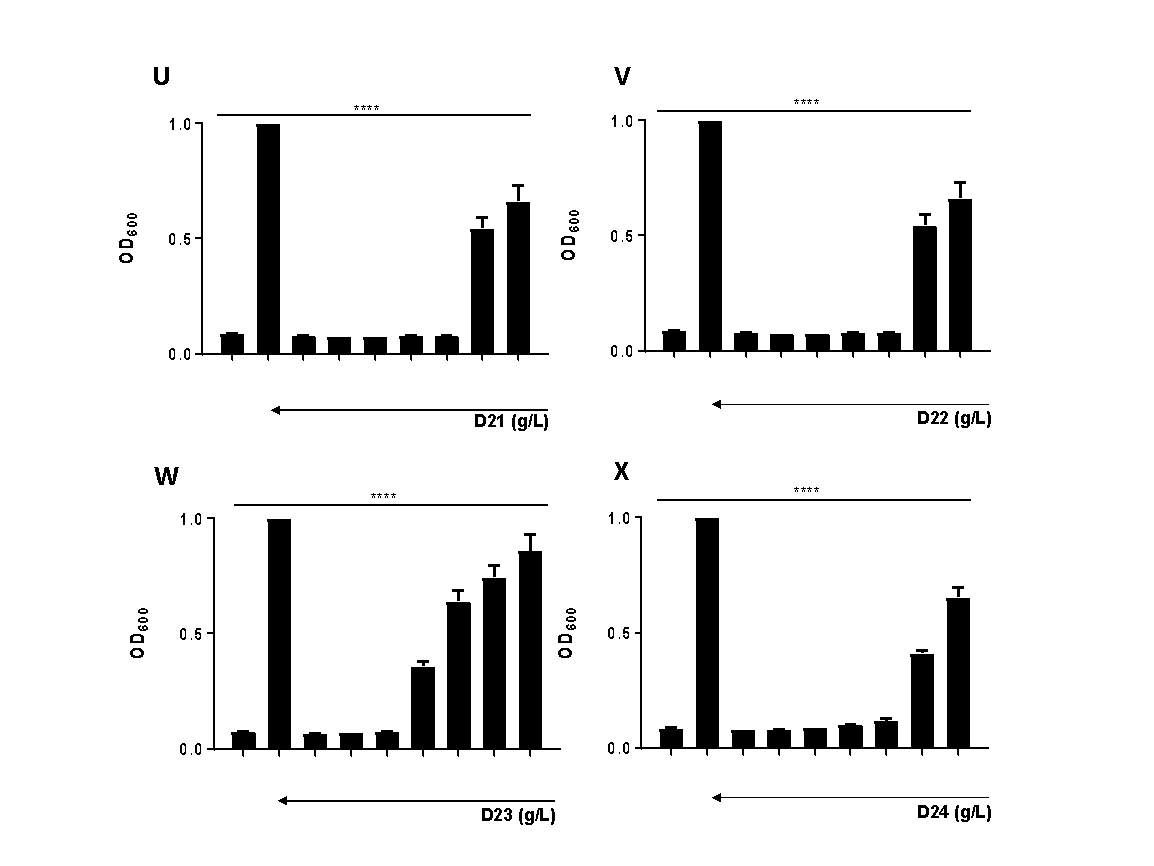


**Figure 1.** Antibacterial activity of 24 disinfectants (A-X) against *E. coli* ATCC 11229 (P-value <0.005). Ampicillin (10ug / mL) was used as a positive control.


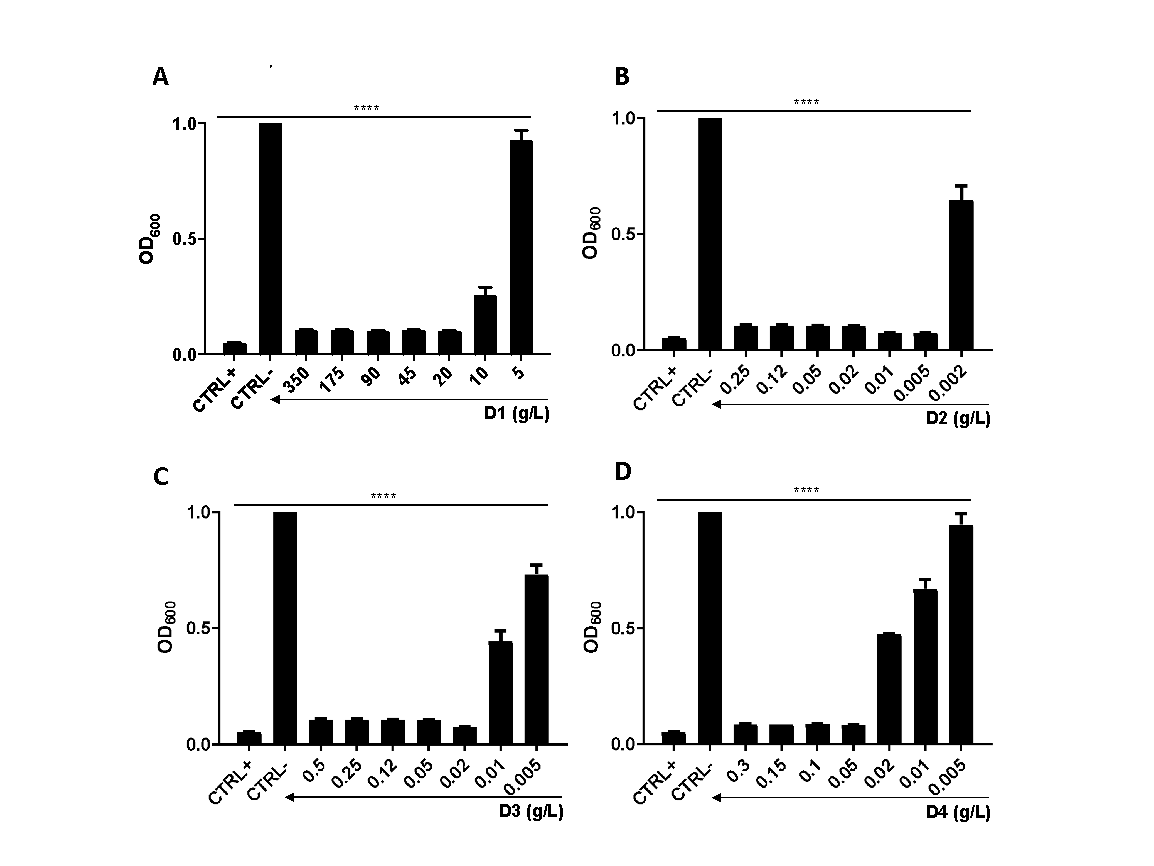

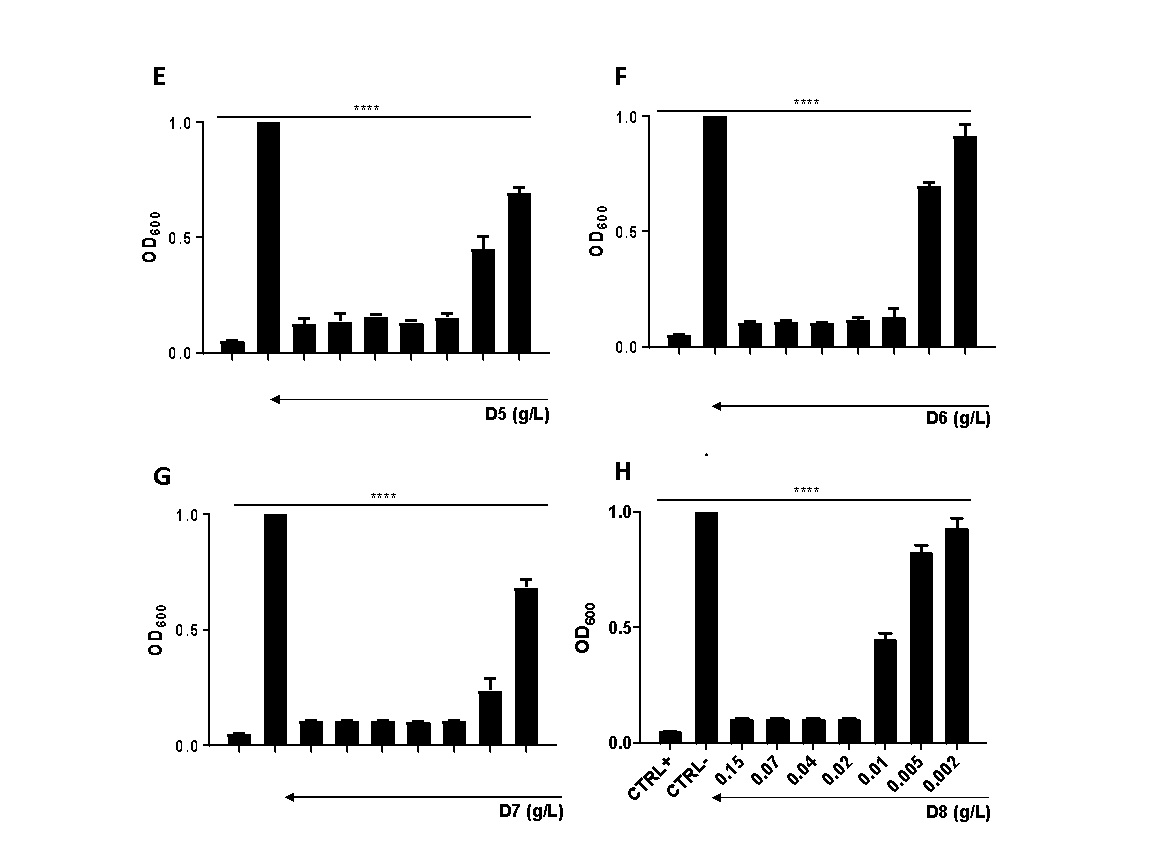

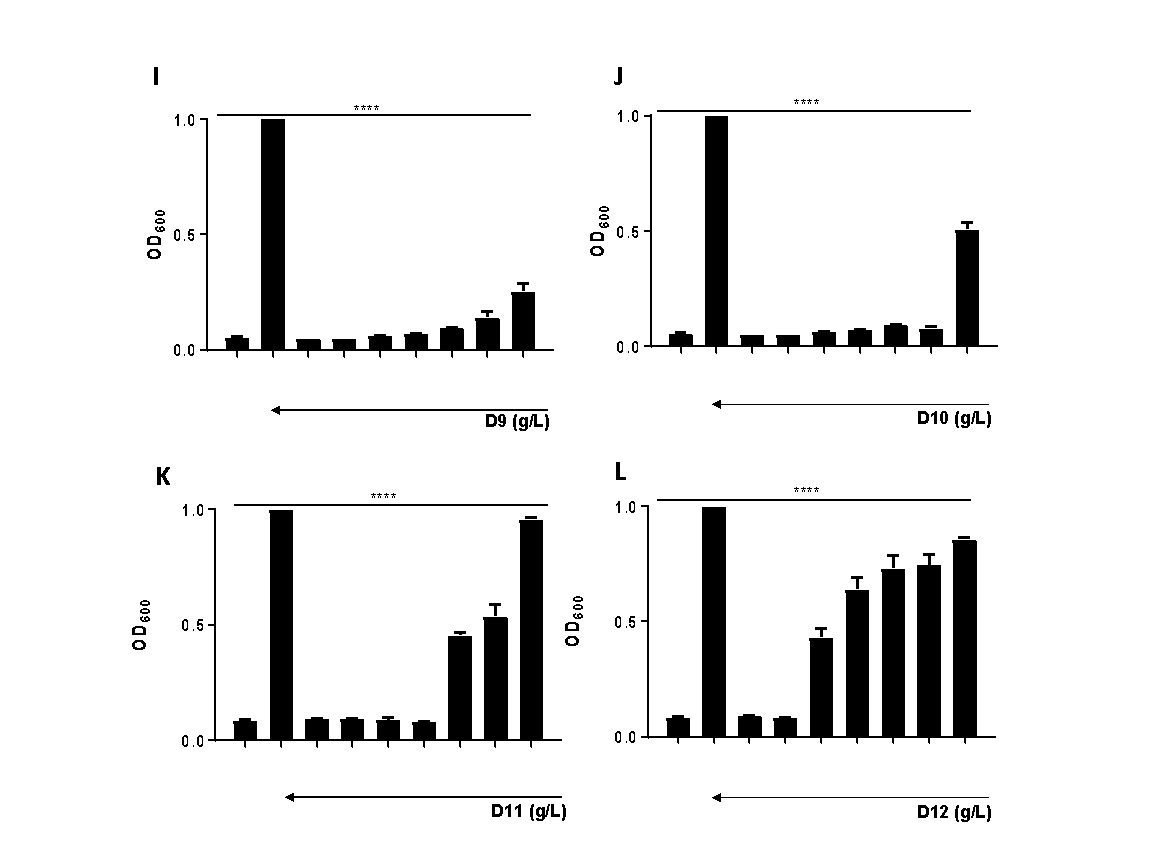

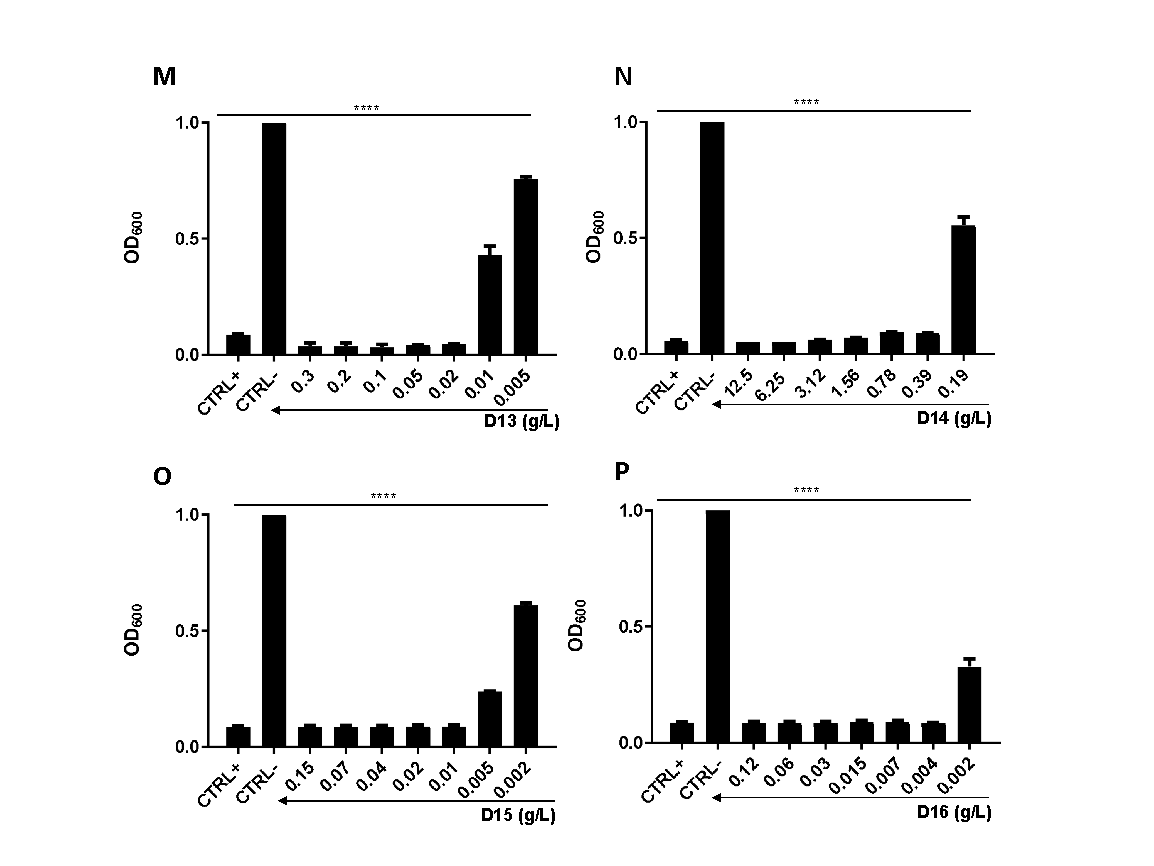

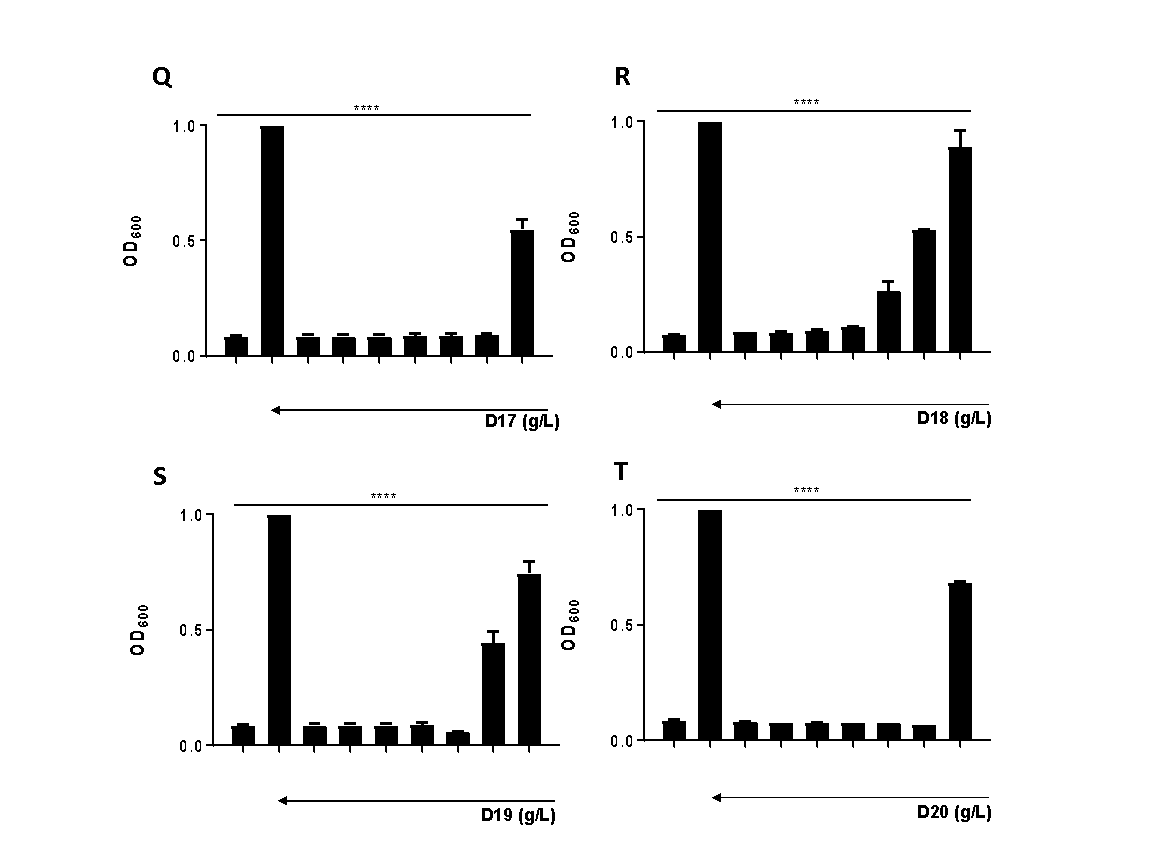

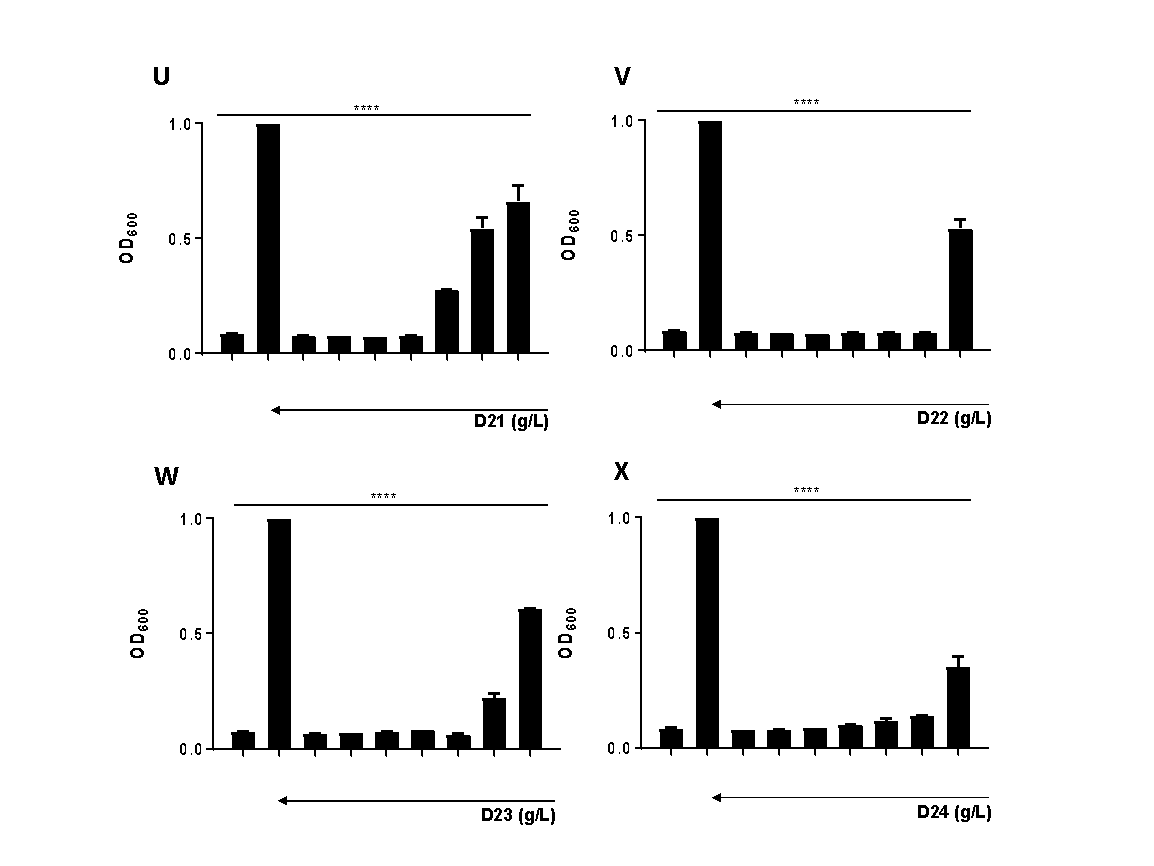


**Figure 2.** Antibacterial activity of 24 disinfectants (A-X) against *S. aureus* ATCC 6538 (P-value <0.005). Vancomycin (20ug / mL) was used as a positive control.


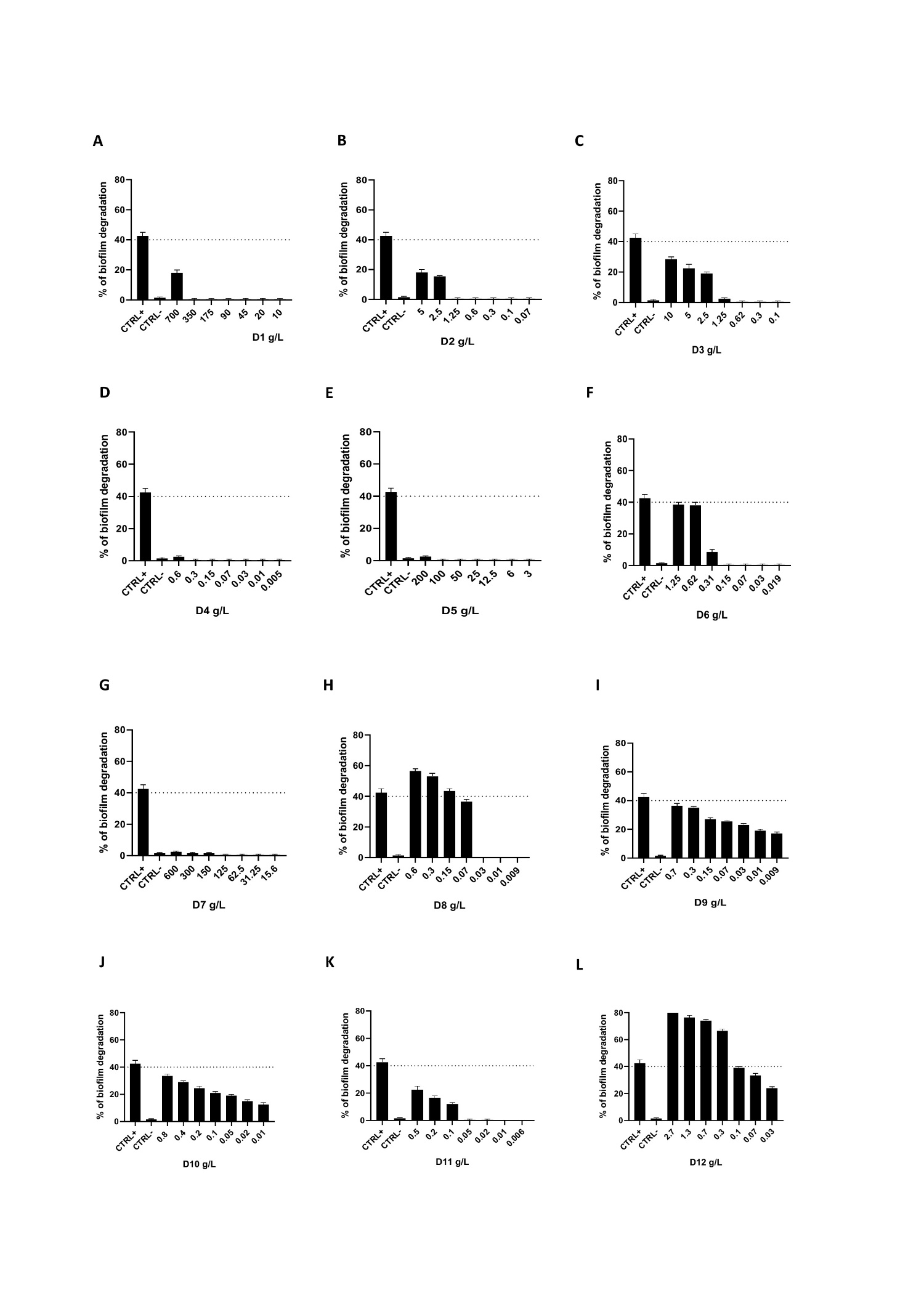


**Figure 3.** Antibiofilm activity of 24 disinfectants (A-X) against *S. aureus* ATCC 6538 (P-value <0.005). Vancomycin (20ug / mL) was used as a positive control.


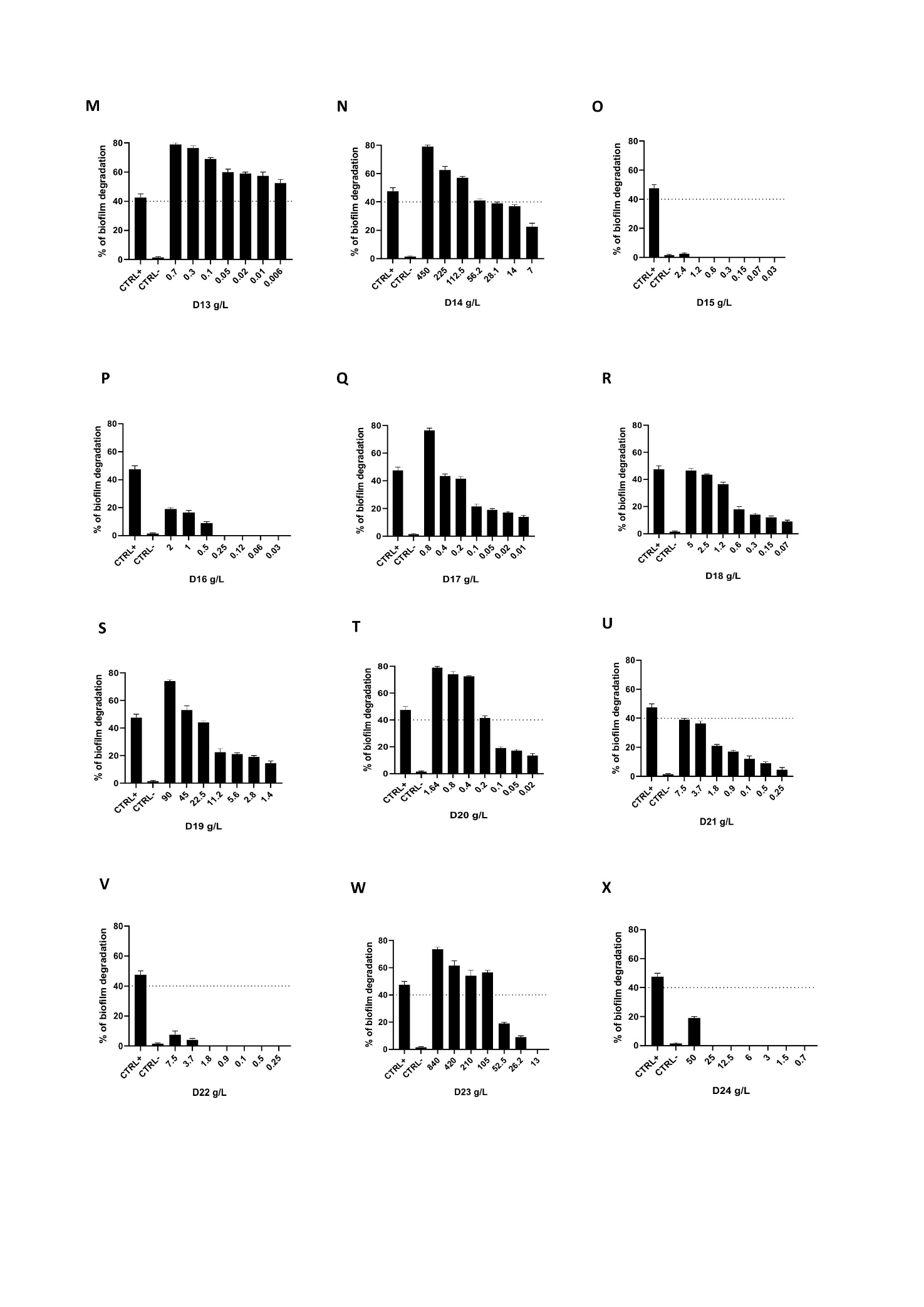


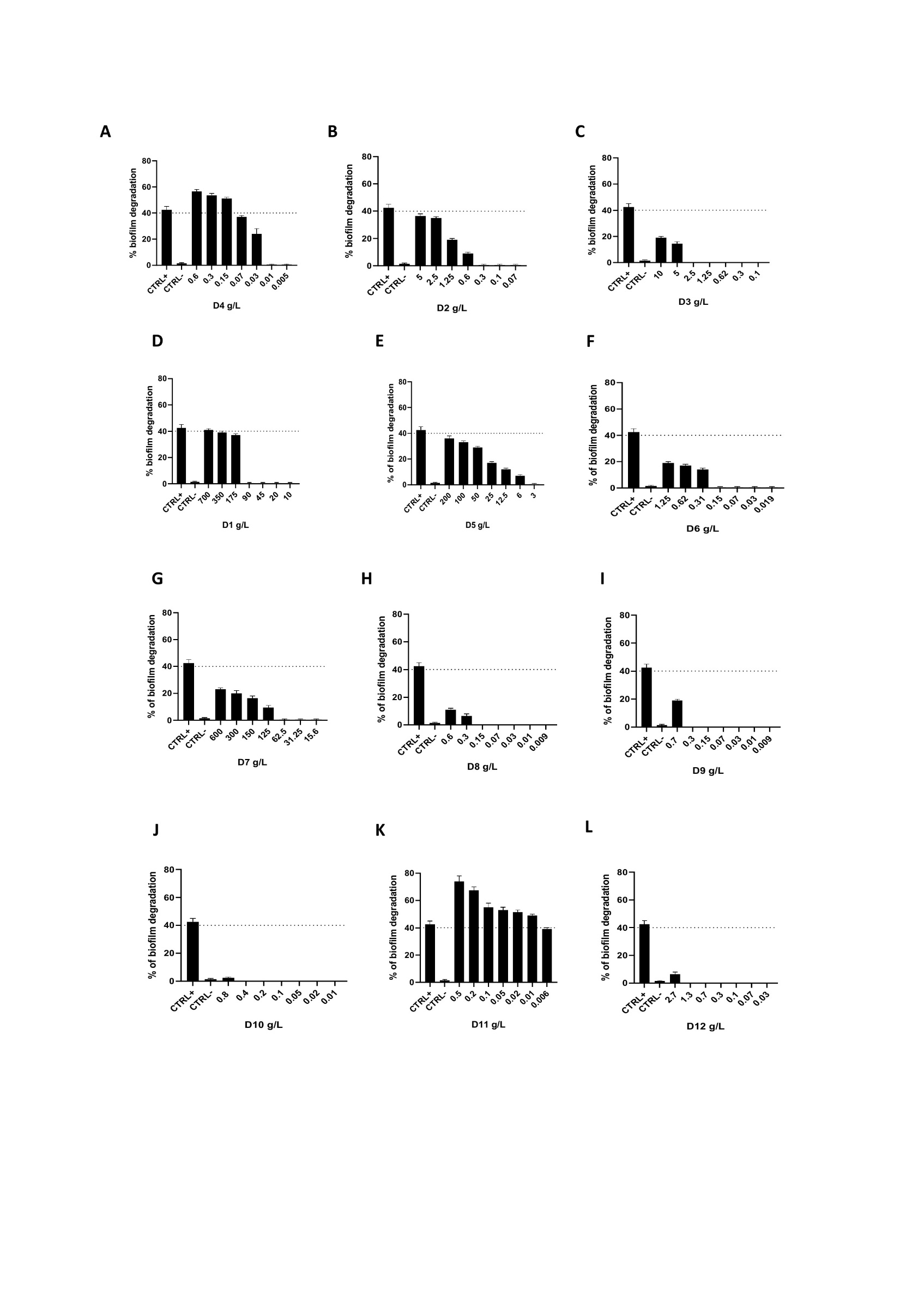


**
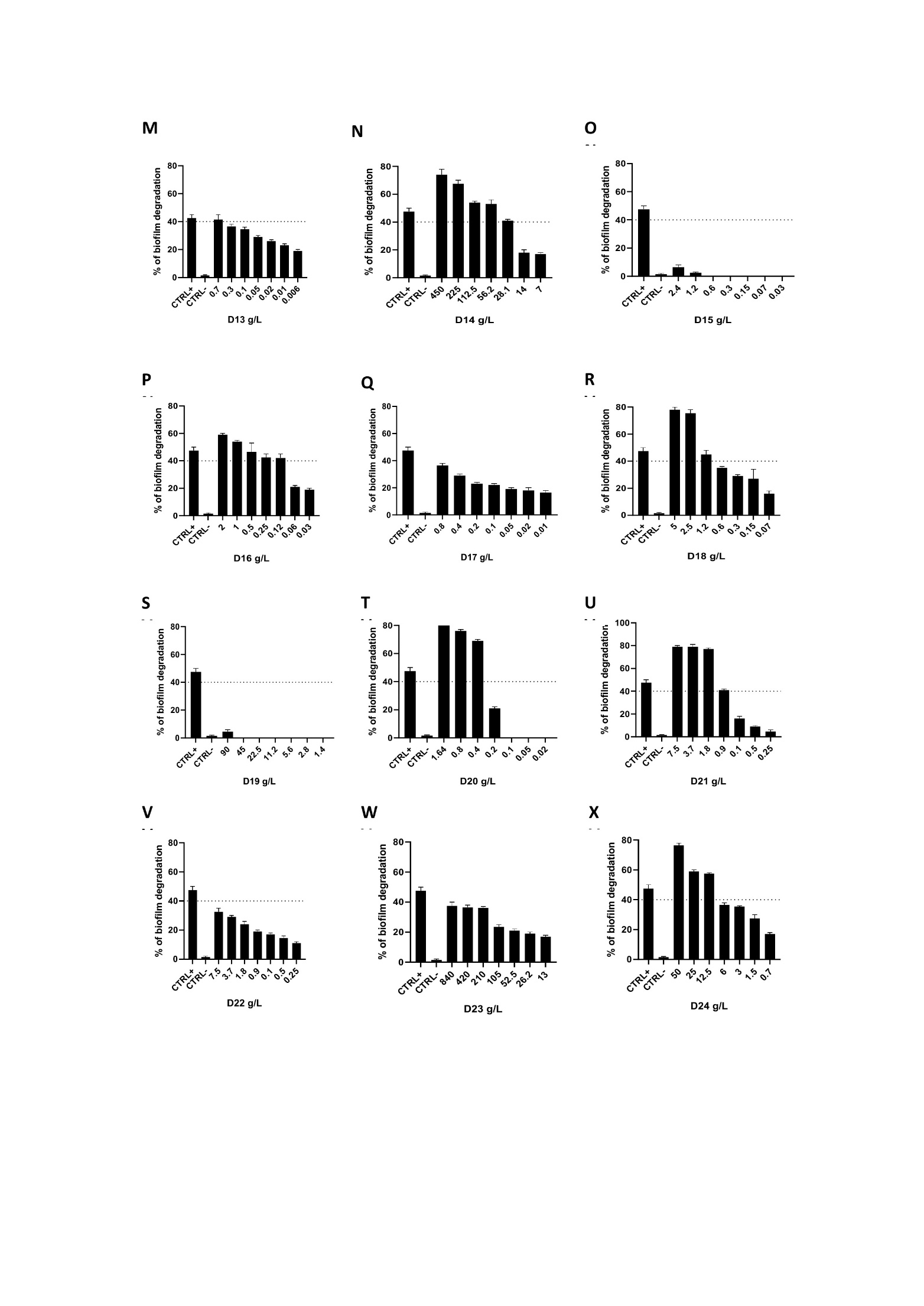
Figure 4.** Antibiofilm activity of 24 disinfectants (A-X) against *E. coli* ATCC 11229 (P-value <0.005). Ampicillin (10ug / mL) was used as a positive control.


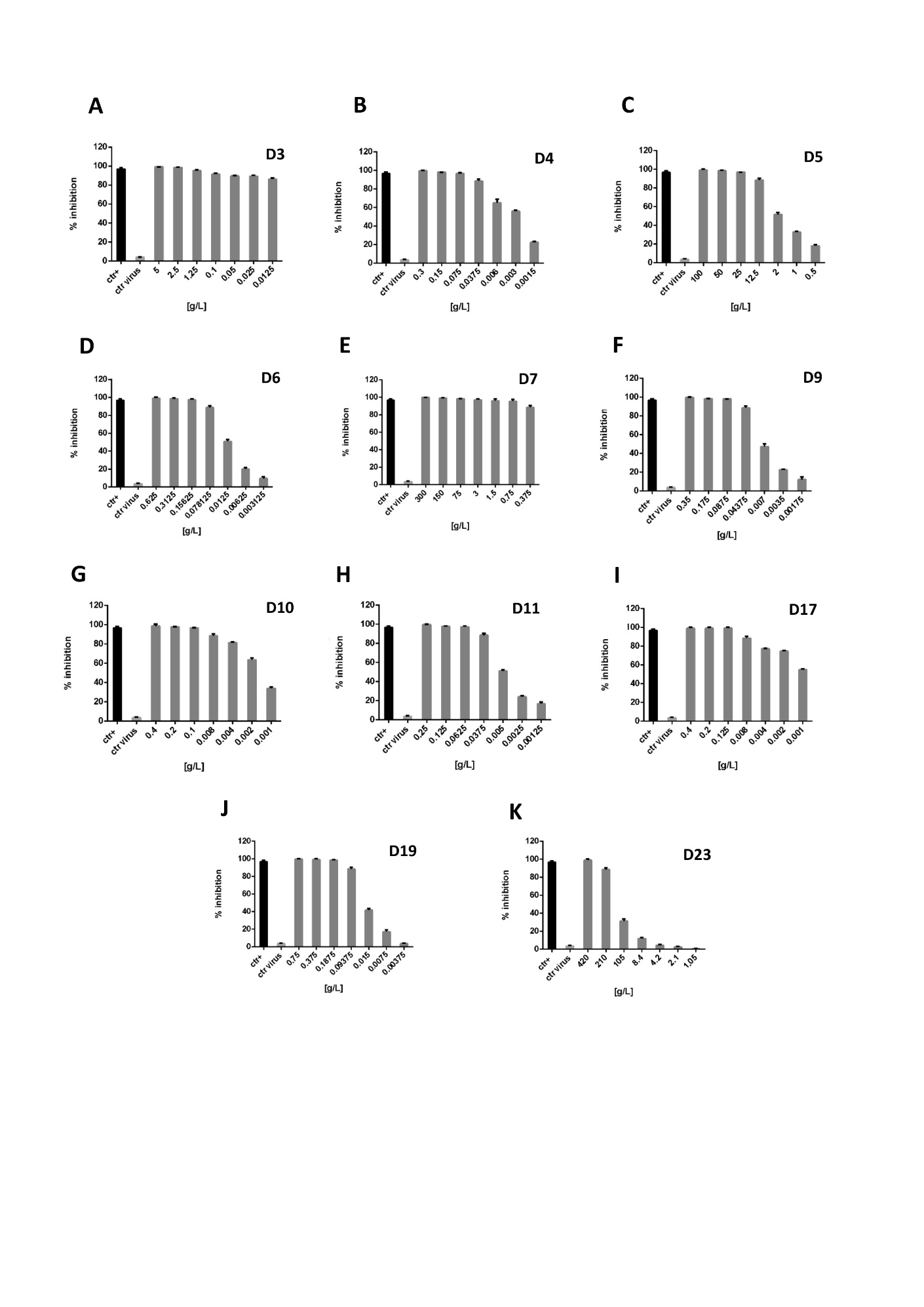
**Figure 5.** Antiviral activity of disinfectants (A-K) against SARS-CoV-2. Rhamnolipids M15RL (50 µg / mL) was used as a positive control.

The datasets used and/or analysed during the current study are available from the corresponding author on reasonable request.
